# The inhibition effect of water on the purification of natural gas with nanoporous graphene membranes

**DOI:** 10.3762/bjnano.9.182

**Published:** 2018-07-02

**Authors:** Krzysztof Nieszporek, Tomasz Pańczyk, Jolanta Nieszporek

**Affiliations:** 1 Department of Theoretical Chemistry, Maria Curie-Sklodowska University, M. C. Sklodowska sq. 3, 20-031 Lublin, Poland; 2 Jerzy Haber Institute of Catalysis and Surface Chemistry, Polish Academy of Sciences, Niezapominajek 8, Krakow, Poland; 3 Department of Analytical Chemistry and Instrumental Analysis, Maria Curie-Sklodowska University, M. C. Sklodowska sq. 3, 20-031 Lublin, Poland

**Keywords:** graphene membrane, hydrogen bonds, molecular dynamics, separation

## Abstract

Molecular dynamics simulations are used to investigate the inhibiting effect of water on the natural gas separation with nanoporous graphene. The membrane separation process involves CH_4_ + N_2_ mixtures with and without the addition of water. The results show that water is able to form hydrogen bonds with nitrogen atoms located in a nanopore rim. This effect causes a decrease of separation selectivity as well as a reduction of gas permeation. In the extreme case, when the nanopore rim contains only nitrogen atoms, water agglomerates at the center of the nanopore and effectively closes down the permeation path. The conclusions are confirmed by the analysis of stability and kinetics of hydrogen bonds.

## Introduction

Modern separation techniques require energy-efficient and environmentally friendly solutions. Very promising, but not yet widely considered to be practical, is the utilization of nanochemistry achievements. An example would be the utilization of nanoporous graphene as a gas separation membrane, because of the high mechanical strength and monoatomic thickness of graphene. Graphene is believed to be the strongest material ever measured [1] with electrical and thermal conductivities higher than those of any other known substance [2].

There are a number of factors to consider regarding graphene as a separation membrane. Commonly applied membranes for gas separation are approximately several micrometers thick. As membrane permeation is inversely proportional to the membrane thickness, nanoporous graphene undoubtedly surpasses the expectations of engineers. While considering production techniques, the application of focused electron beam irradiation in transmission electron microscopy makes it possible to determine graphene membranes with well-defined pore diameter [3]. Similar possibilities are also created by modified helium ion microscopy for lithography [4] and surface-assisted aryl–aryl coupling reactions [5]. Girit et al. [6] observed experimentally the movement of individual atoms at the edge of a graphene hole.

Coal, crude oil and natural gas are the very useful energy sources. Natural gas is commonly assumed to be an environmentally friendly fuel because its combustion products are water and carbon dioxide. However, raw natural gas contains many impurity components, e.g., water, nitrogen and hydrogen sulfide. Thus, before the gas is supplied to the recipient, it must be purified using, e.g., membrane separation. For the above mentioned reasons, the application of nanoporous graphene membrane deserves special attention.

The separation through graphene crucially depends on the method of nanopore passivation. There are a lot of papers on graphene membranes doped with hydrogen, nitrogen or oxygen [7–9]. Sakaushi and Antonietti wrote an interesting review on carbon- and nitrogen-based materials [10]. Hauser and Schwerdtfeger [11] studied theoretically the ability of functionalized graphene nanopores to separate methane from air. Recently, Guerrero-Avilés and Orellana performed ab initio MD simulations to study the interactions between hydrated ions and liquid water with porous graphene [12]. Sun et al. [7] studied the purification of natural gas using nanoporous graphene with the help of classical molecular dynamics. Similarly to most papers that deal with the application of nanoporous graphene, they demonstrated that the efficiency of separation relies strongly on the pore size and geometry. The presence of a strongly electronegative atom in the way of separated components may cause additional, probably undesirable effects. For example, during natural gas separation, the water molecules, which are present in the mixture, can lower the permeation rate due to hydrogen-bonding with the nanopore rim. In this paper, by means of classical molecular dynamics, we discuss such an inhibiting effect of water on the gas permeability. Employing different nanopores functionalized by hydrogen and nitrogen atoms, we conduct theoretical studies on the separation capability of graphene membranes for natural gas mixtures. The phenomenon of clustering of water in the vicinity of nanopores, is clearly visible in all separation systems including the nanopores functionalized by nitrogen atoms. Although this paper is purely theoretical, the authors believe that the results are valuable for practical purposes and can be applied to design real membrane-separation systems.

## Simulation Model

**Figure 1:**
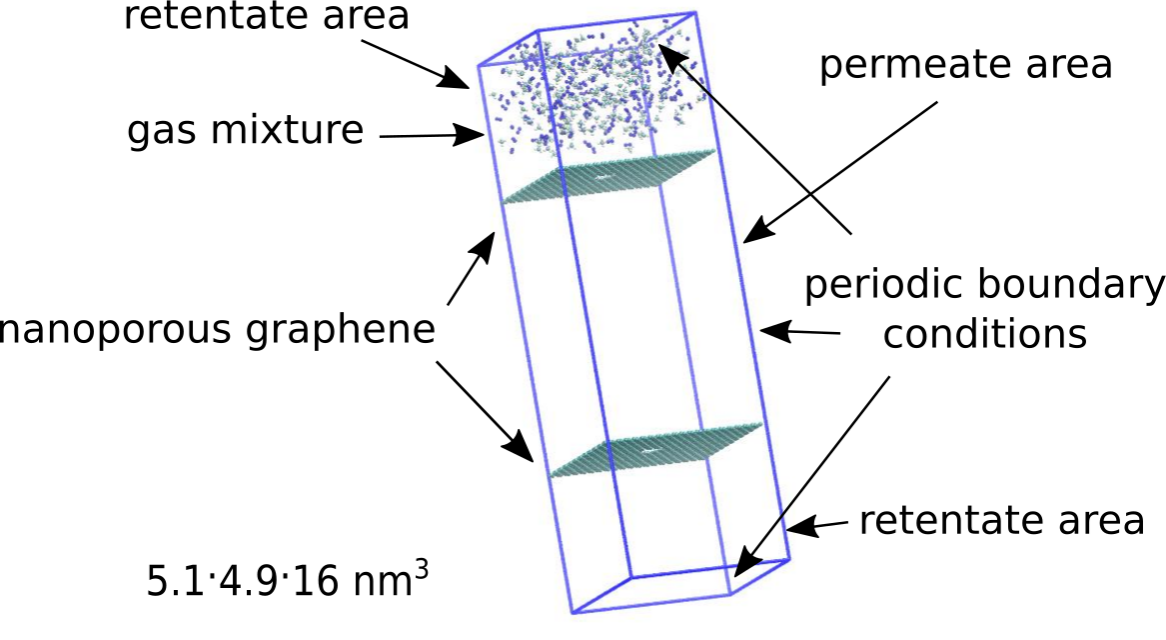
The simulation box.

The simulation system chosen by the authors is similar to that used in [13]. In brief, all calculations were conducted using Gromacs 4.6.7 suite [14] in NVT ensemble employing the OPLS all-atom force field. The reliability of the applied force field has already been verified [9,16–17] and shows a good agreement with experimental as well as theoretical data [18]. Simulations were performed in a cuboid box of 5.1 nm × 4.9 nm × 16 nm with two graphene sheets located at *z* = 4 nm and *z* = 12 nm dividing the box into two chambers. Retentate areas were above and below the graphene sheets, whereas the permeate area was between the graphene sheets in the range of 4 nm < *z* < 12 nm (Figure 1). Periodic boundary conditions were applied in all three directions. The temperature was maintained at 300 K using velocity rescaling with a stochastic term [19] and a time constant of 0.1 ps. In order to enable possible deformations, the graphene sheets were flexible; to avoid vertical dislocation due to collisions with separated gases, the carbon atoms in each graphene corner were fixed. Cutoff distances for Lennard-Jones and Columbic interactions were set to 1 nm and equations of motion were integrated using the velocity Verlet algorithm with a time step of 0.5 fs. Each simulation was equilibrated for 1 ps and then run for 10 ns.

**Figure 2:**
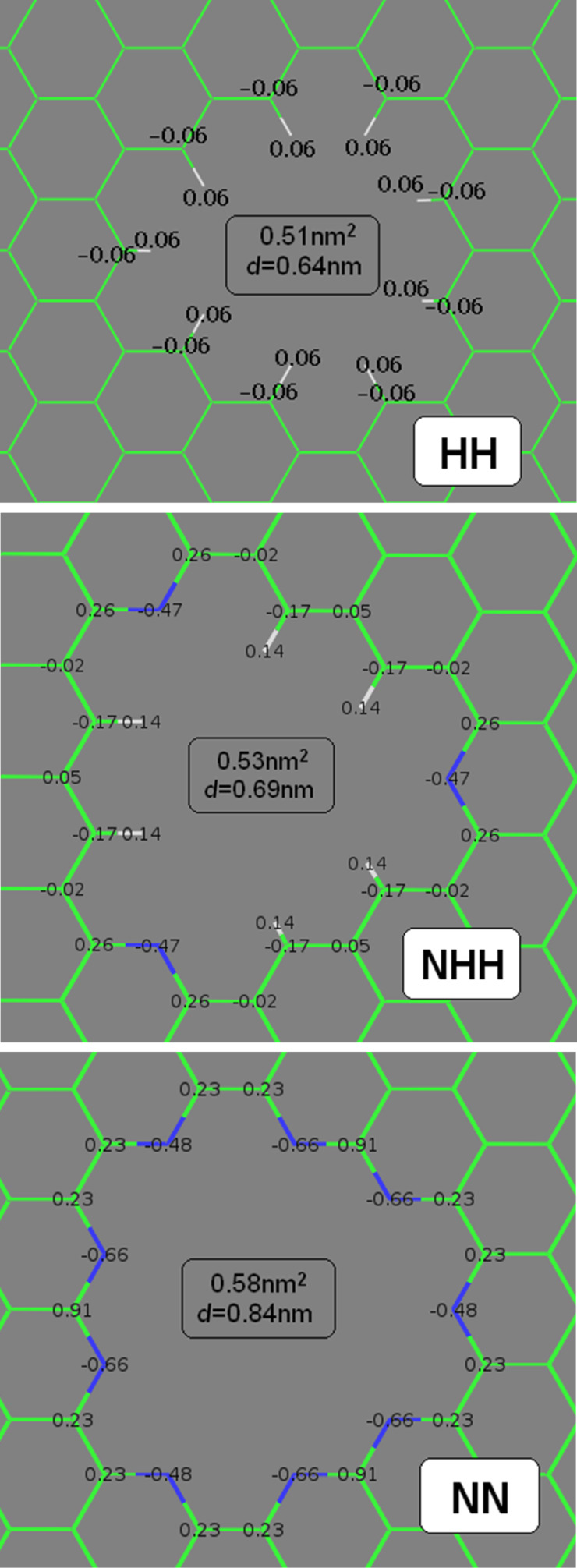
Atomic charges for nanopores HH (top), NHH (middle) and NN (bottom). The charges of the remaining carbon atoms in the graphene sheets were assumed to be zero. The charges of hydrogen atoms in the nanopore HH were obtained using the OPLS force field. Average pore areas and diameters are denoted in the figure.

In practice, it is very challenging to produce highly uniform pores on graphene membranes with the help of commonly used plasma or ion bombarding methods. Thus, the simulations should give an idea of molecular-level phenomena under various conditions. In the present paper three functionalized nanopores were used. Their geometry is similar to the ones used by Sun and co-workers [7]. Therefore, the presented studies can be referred to results that have already been published. Moreover, the nanopores employed here are similar in size to the ones designed by other authors studying the separation of simple gases by porous graphene [8–9,20] and are only a bit larger than those created in the experiment [21]. The first nanopore (denoted as nanopore HH) was created by removing 12 benzene rings from the graphene sheet and adding single hydrogen atoms to every carbon in the pore rim (the pore is H-passivated). The second pore NHH was obtained by replacing three triangularly distributed carbon atoms with nitrogen atoms in the pore rim (the pore rim includes two hydrogen atoms and one nitrogen atom in sequence). The last nanopore (denoted as nanopore NN) was prepared by replacing all carbon atoms (to be precise all ≡C–H groups) in the rim with nitrogen atoms. A variable degree of pore functionalization will be helpful to investigate the influence of water on the gas permeation through the nanopores. Atomic charges for atoms in the rim of nanopore HH were obtained using the OPLS force field, whereas the charges for atoms in the rims of nanopores NHH and NN were obtained by R.E.D. Server [22] using the RESP-A1A (HF/6-31G*) charge model and the Gaussian 09 quantum mechanics program (Figure 2).

MD simulations were performed by putting a CH_4_ + N_2_ gas mixture composed of 200 + 200 molecules and a varying amount of TIP3P water (0 and 200 molecules) in the retentate area. Although Figure 1 shows that at the beginning the gaseous mixture is located only in the upper part of the simulation box, it diffuses to the bottom part of the retentate area due to the presence of periodic walls just after several simulation steps. Such a starting configuration causes an initial pressure difference between the feed side (below and above graphene sheets, see Figure 1) and the permeating area. Therefore, the increase of entropy is the driving force of the process. It is worth mentioning that the presence of 400 molecules (CH_4_ + N_2_) in the retentate area at 300 K corresponds to a pressure of about 84 bar (1200 psi). We decided to use such high pressure because of the fact that the typical pressure of natural gas transported in pipelines is from 200 to 1500 psi. Moreover, an increase of the number of molecules at the feed side significantly shortens the simulation times.

While looking for hydrogen bonds between water and the nanopore, we adopted widely used geometric criteria: *R* < 0.35 nm and 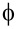 < 30° (please see Laage and Hynes [15]); where *R* is the O_w_^…^N distance and 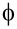 is the angle between O_w_–H and O_w_–N (O_w_ means an oxygen atom in a water molecule, and N is a nitrogen atom located in the nanopore rim).

## Results and Discussion

Molecules can migrate through the nanopores from the retentate to the permeate area. The observed number of molecules passing across the pores is different for methane and nitrogen because of different kinetic properties of the gases as well as the interaction energy with the nanopores. Moreover, adsorption effects play a crucial role in separation processes [13]: The permeating molecule can get stuck in the center of nanopore and cause a decrease of flux of the second mixture component. Such a case can be observed when the separated mixture includes water or other hydrogen-bond donors and the graphene nanopore rim is passivated by strongly electronegative atoms such as oxygen or nitrogen. As a result, the nanopore radius can be reduced due to hydrogen bonds between water and nitrogen. Therefore, to study such an effect we have chosen three nanopores that vary in the ability to form hydrogen bonds with water molecules.

**Figure 3:**
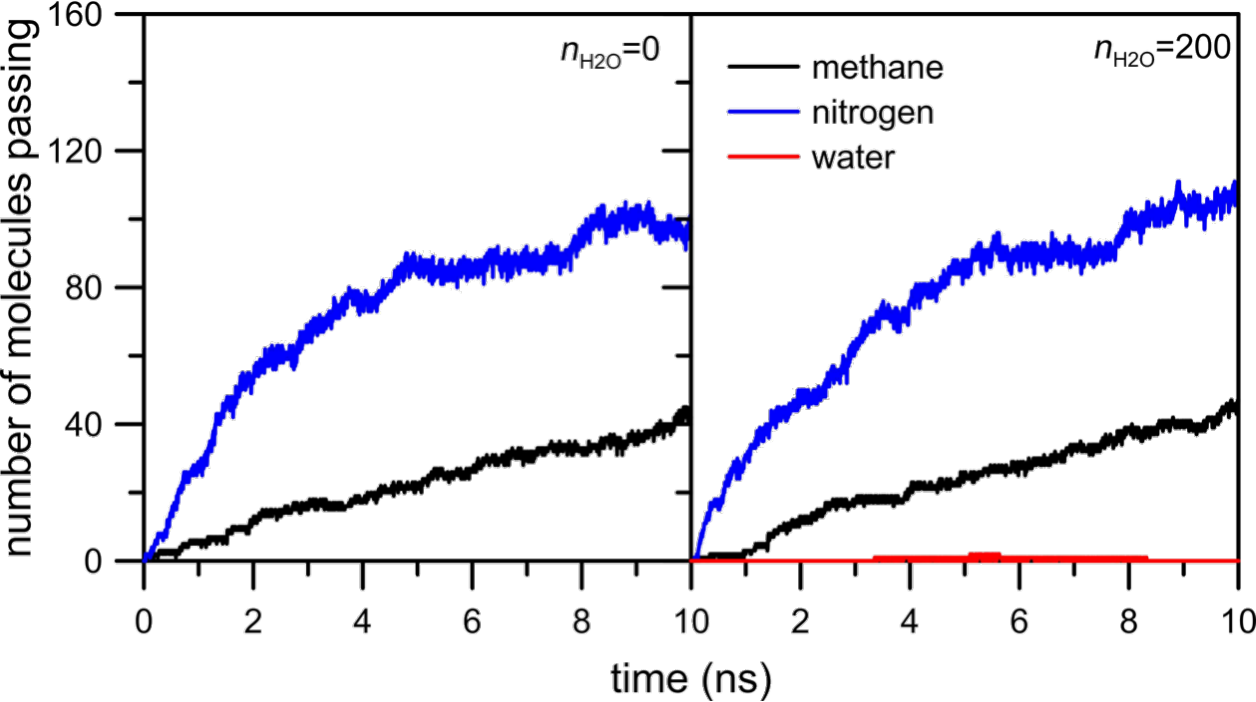
The influence of water on the number of methane and nitrogen molecules passing through an HH nanopore.

The first simulation system under investigation includes a membrane with hydrogen atoms at the pore rim. Figure 3 shows the influence of water on the permeation rate of CH_4_ + N_2_ gaseous mixture across an HH nanopore.

It can be seen that the presence of water in the retentate area does not visibly affect the number of methane and nitrogen molecules passing through the HH nanopore. This is because the nanopore includes only hydrogen atoms, which are weak hydrogen bond (HB) donors in this system. Thus, the water molecules can influence the permeation rate through HH nanopore only through steric effects. Moreover, the observed number of water molecules passing the pore is 1 or 2. The careful analysis of the animation video of the simulations shows that water atoms form a big drop at the graphene surface. Hence, the water–water interactions stop the water in the retentate area.

**Figure 4:**
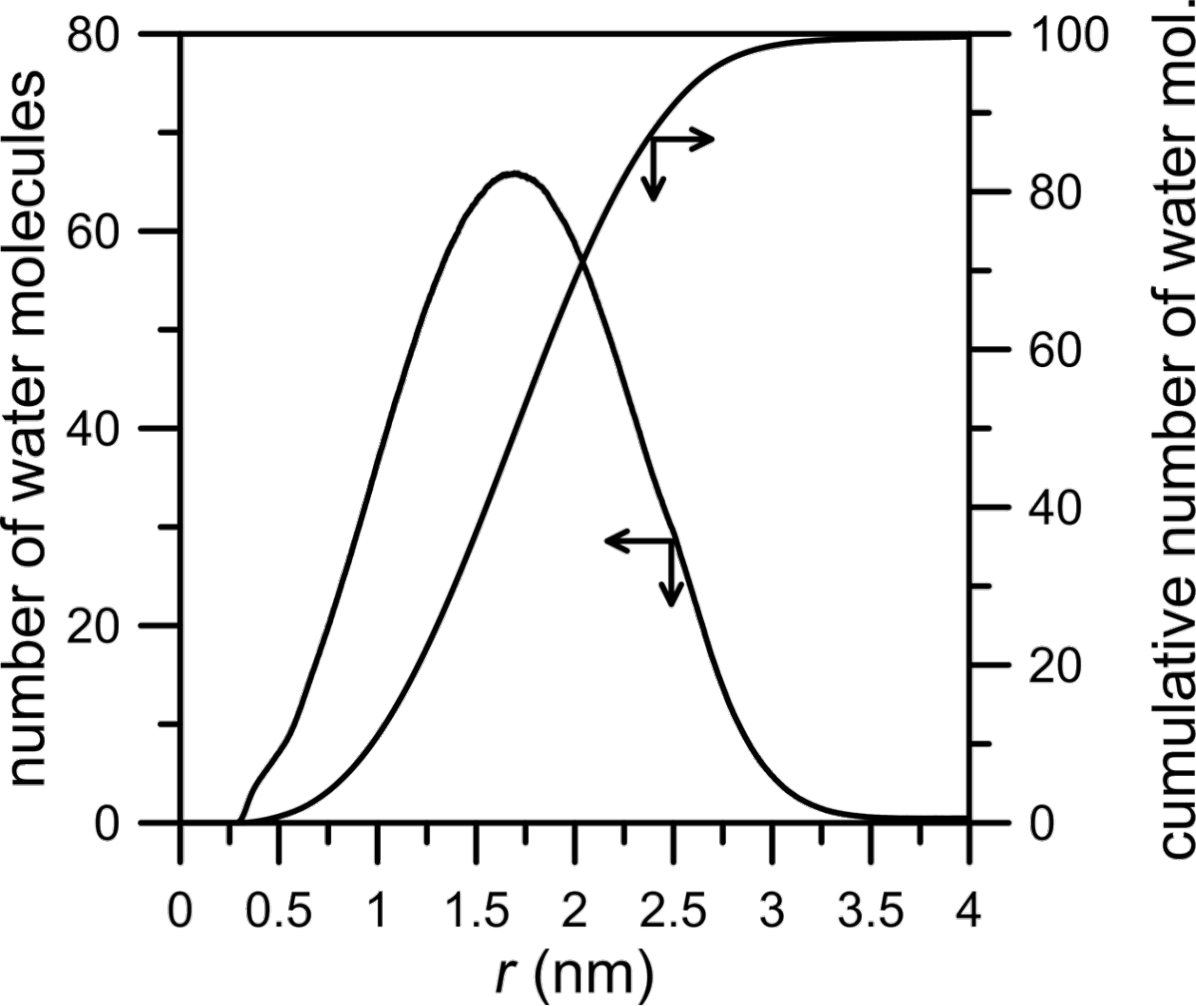
The average number of water molecules as a function of the distance of O_w_ atoms from the center of the rim of an HH nanopore calculated for a CH_4_ + N_2_ gaseous mixture containing 200 water molecules. The “cumulative number of water molecules” means the average number of particles within a distance *r*, whereas the “number of molecules” is the non-normalized radial distribution function (RDF), e.g., the RDF without dividing the probability density by the average density in the whole box. The bin width is 0.002 nm.

The conclusion above can be confirmed by the number of water molecules (NWM) within a given distance from the nanopore center (calculated as the distance of O_w_ atoms from the geometric center of carbon atoms at the edge of the nanopore rim) and the cumulative number of water molecules within this region shown in Figure 4.

It is worth mentioning that the average HH nanopore diameter is 0.6 nm and the curves in Figure 4 are plotted as the functions of the distance of O_w_ from the nanopore center. It can be seen in Figure 4 that the peak with a maximum at 1.7 nm indicates a high water density relatively far from the edge of the nanopore. Moreover, the width of the peak indicates that the water is spread out in a wide range around the pore, at the distance of 2.5 nm from the rim the cumulative number of water molecules is about 80.

**Figure 5:**
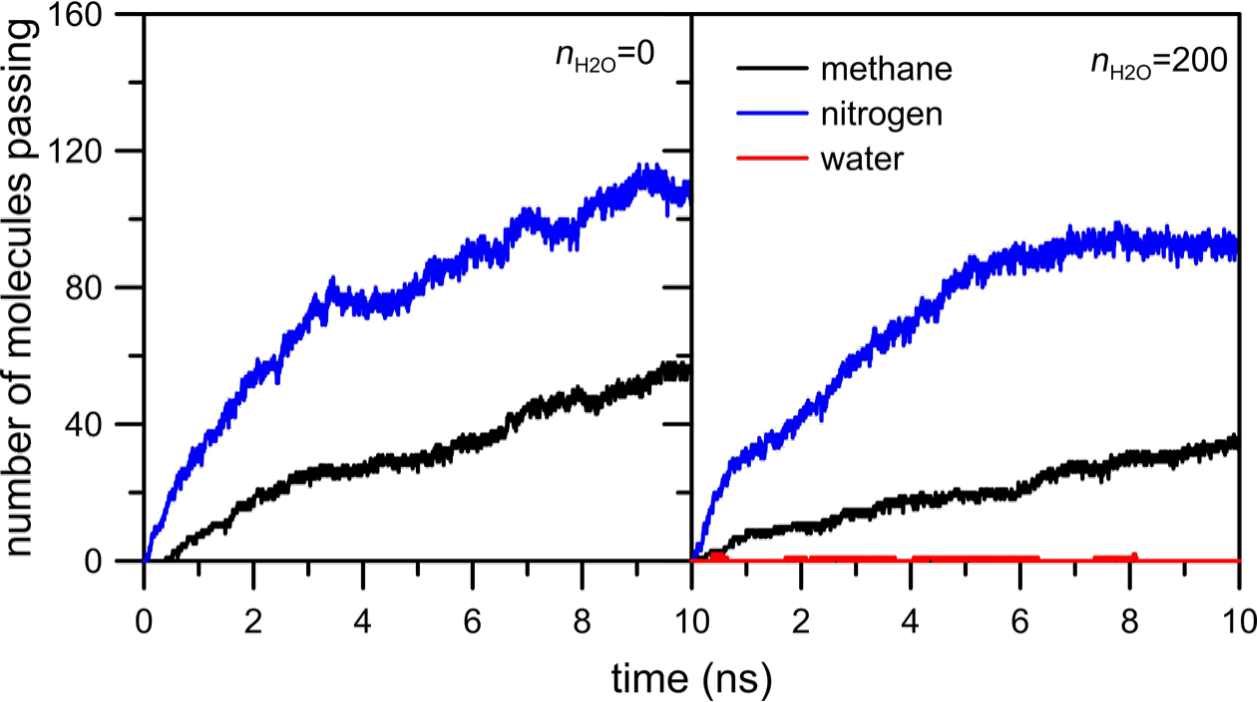
The influence of water on the number of methane and nitrogen molecules passing through an NHH nanopore.

**Figure 6:**
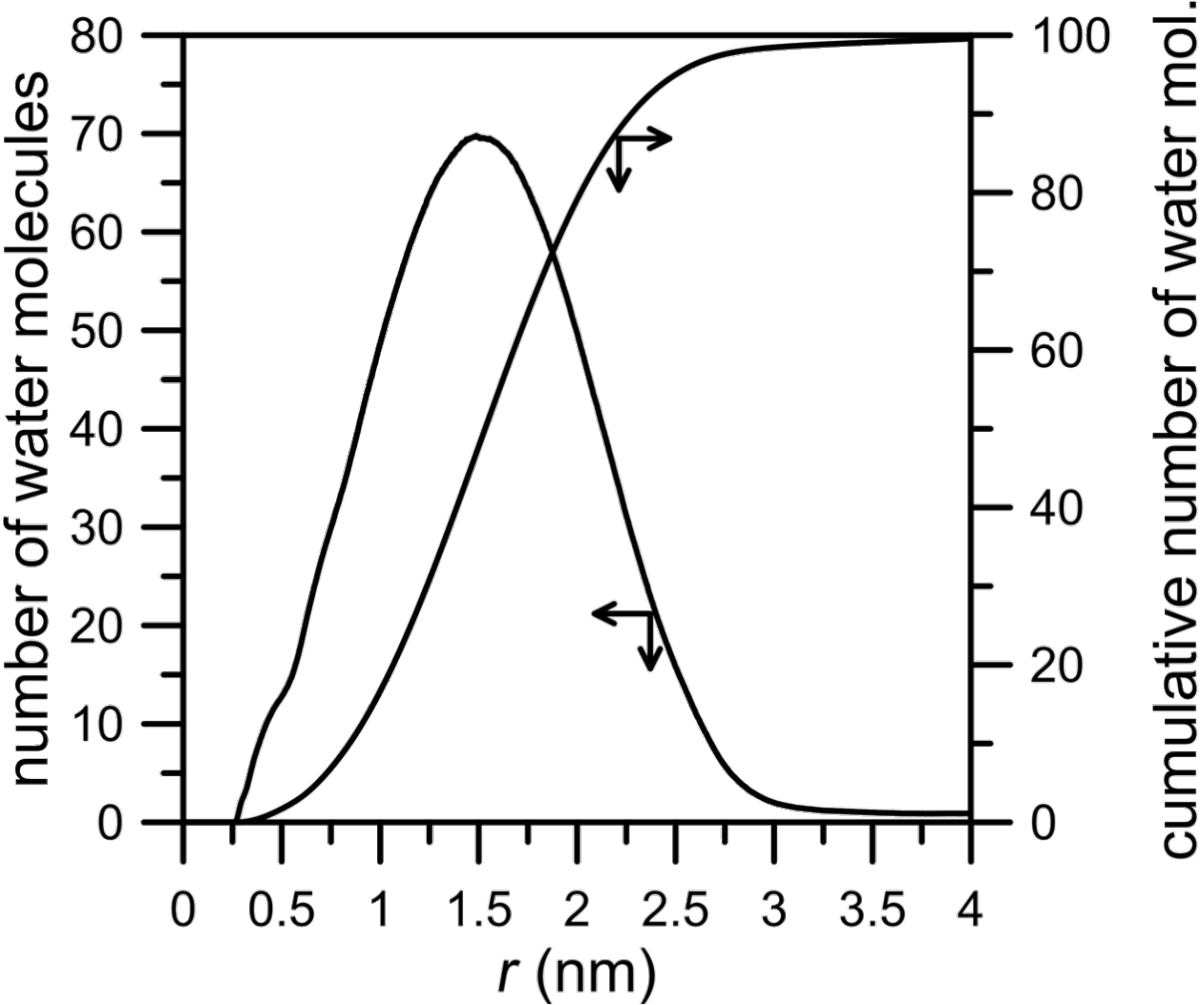
The number of water molecules plotted as a function of the distance of O_w_ atoms from the center of the rim of an NHH nanopore (here it is the geometric center of carbon and nitrogen atoms in the nanopore rim) calculated for a CH_4_ + N_2_ gaseous mixture containing 200 water molecules. Other notes are the same as in Figure 4.

Figure 5 shows the number of CH_4_, N_2_ and H_2_O molecules passing through an NHH nanopore. In the absence of water, the number of nitrogen and methane molecules in the permeate chamber is slightly larger in comparison to the case when water is present in the gas mixture. Like in the previously discussed system, water does not actually permeate through the nanopore. The inhibiting effect of water on the permeation rate is more visible in the case of methane (Figure 5, black line). It can also be observed in Figure 6 in which the peak of NWM is at about 1.5 nm, i.e., in comparison to an HH nanopore the maximum of the NWM peak is shifted towards the pore. The cumulative number of H_2_O molecules at 2.5 nm is about 90.

**Figure 7:**
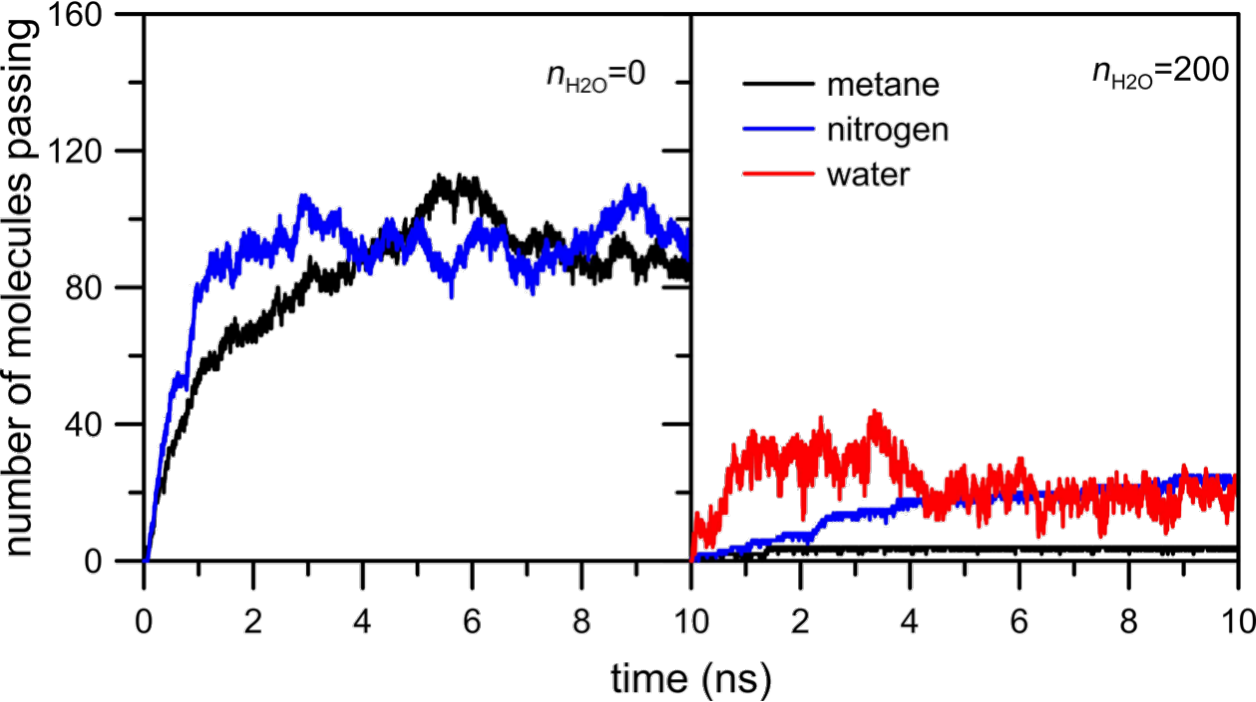
The influence of water on the number of methane and nitrogen molecules passing through NN nanopore.

**Figure 8:**
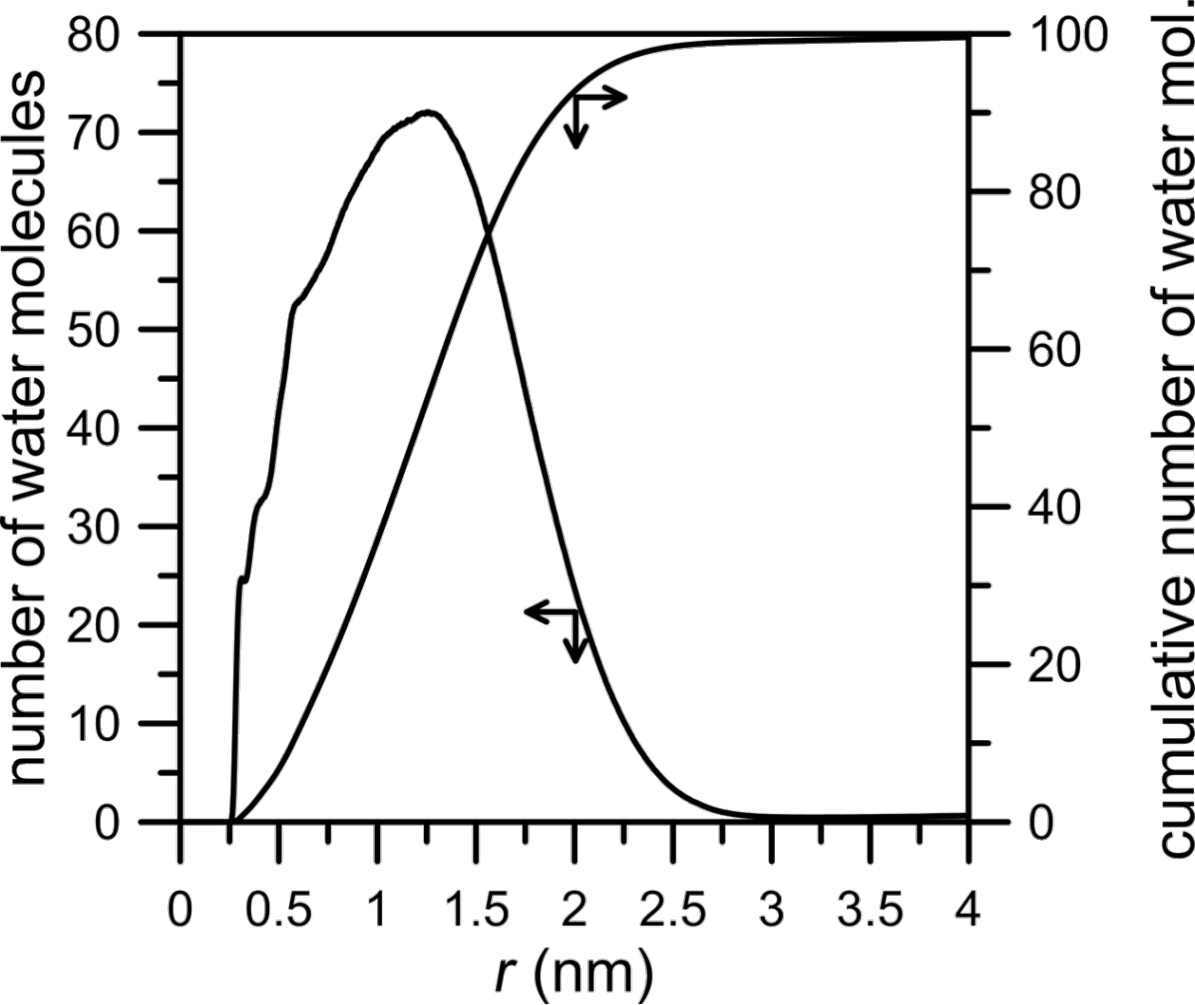
The number of water molecules plotted as a function of the distance of O_w_ atoms from the center of the rim of an NN nanopore (defined as the geometric center of nitrogen atoms in the nanopore rim) calculated for a CH_4_ + N_2_ gaseous mixture containing 200 water molecules. Other notes are the same as in Figure 4.

The last case to consider concerns the permeation through a nanopore with the rim passivated entirely by nitrogen atoms. Figure 7 and Figure 8 clearly show the inhibiting effect of water on the permeation rates of methane and nitrogen. The number of molecules passing across the pore decreases for both gases. Still, the number of water particles observed in the permeate area is below 20. The NWM function (Figure 8) shows that most water molecules are gathered close to the nanopore. The big water drop almost completely blocks the permeation path. At a distance of 0.5 nm from the nanopore center the amount of water molecules is about 40. It is worth comparing Figure 8 with Figure 4 and Figure 6 – the hydrogen bonds between water presented in the purified mixture and the nanopore rim can play a substantial role in the separation process. Thus, such an effect should be taken into account when designing separation systems.

Figure 7 indicates that the effect of moisture on the reduction in the flow rate of the two gases is different. It is probable that the flow rate of methane is affected more than that of nitrogen due to the size difference between the two gas molecules. Moreover, the comparison of Figure 3, Figure 5 and Figure 7 shows a sharp increase of the number of passings for both molecules when the pore is fully passivated by nitrogen atoms even in the absence of water molecules. Such an interesting and peculiar behavior of the NN pore results from the fact that nitrogen passivation is the replacement of ≡C–H groups in the nanopore rim with nitrogen atoms. When the pore is totally or partially H-passivated, carbon atoms in the graphene hole are bonded with hydrogens. As a result, the effective diameter of the NN nanopore is a bit larger than that in the other considered cases.

**Figure 9:**
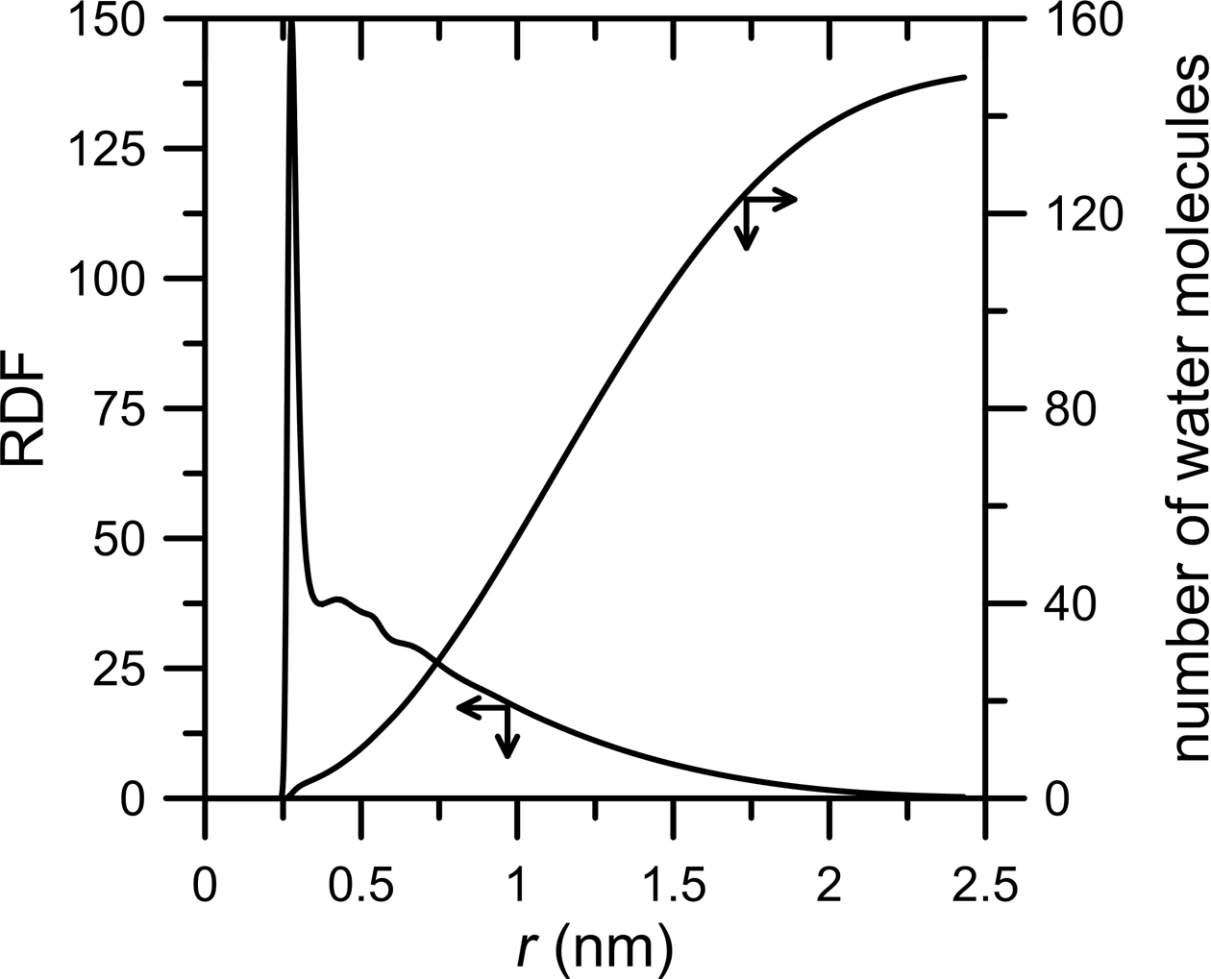
Radial distribution function of O_w_–O_w_ pairs and the cumulative number of water molecules for the NN membrane system and CH_4_ + N_2_ gaseous mixture containing 200 water molecules.

The hyphotesis concerning the big water drop in the NN nanopore can be confirmed by the RDF function of a O_w_–O_w_ pair. Figure 9 shows that the majority of water molecules are close together.

**Figure 10:**
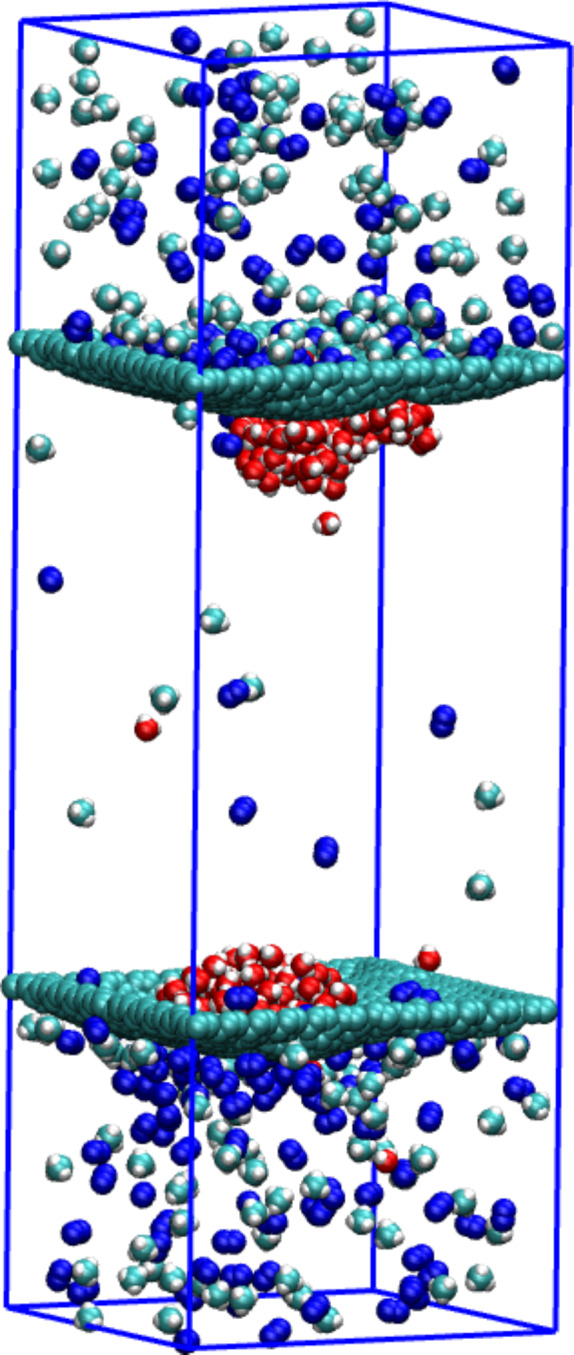
The extracted frame from the simulation trajectory of the separation of CH_4_ + N_2_ + 200 H_2_O with an NN nanopore at a time of 5 ns. Green, blue and red molecules are methane, nitrogen and water, respectively.

An illustration of the clustering of water molecules in the NN nanopore can be seen in Figure 10. The picture has been extracted from the simulation trajectory at a time of 5 ns. In Figure 7 (right panel, red curve) an attentive reader can see a decrease of the number of water molecules in the permeate area at a time of ca. 4 ns. It looks like a reflux of water molecules from the permeate to the retentate area. Nevertheless, the picture in Figure 10 shows that water still forms a drop. Thus, some water molecules can migrate through the nanopore but still stay in its close vicinity.

**Figure 11:**
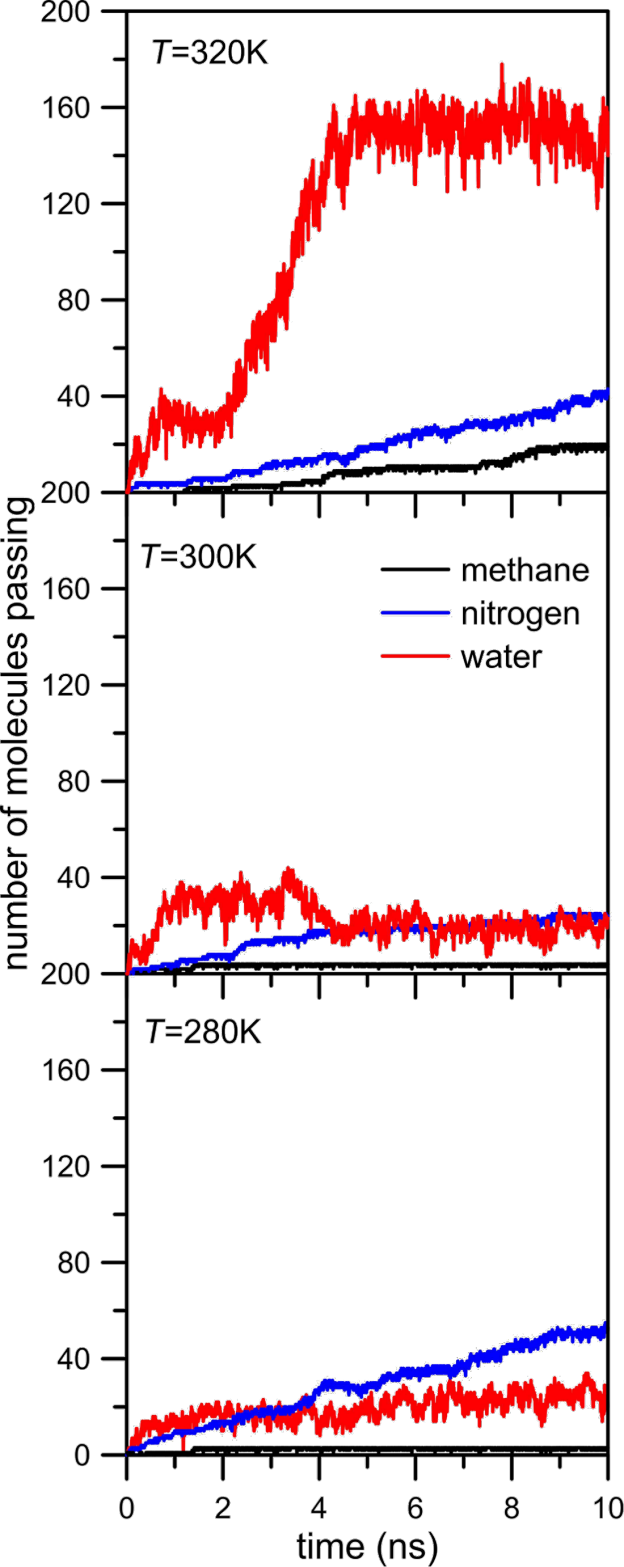
The temperature influence on the number of molecules passing across the pore as a function of the simulation time for CH_4_ + N_2_ + 200 H_2_O separation with an NN nanopore.

**Figure 12:**
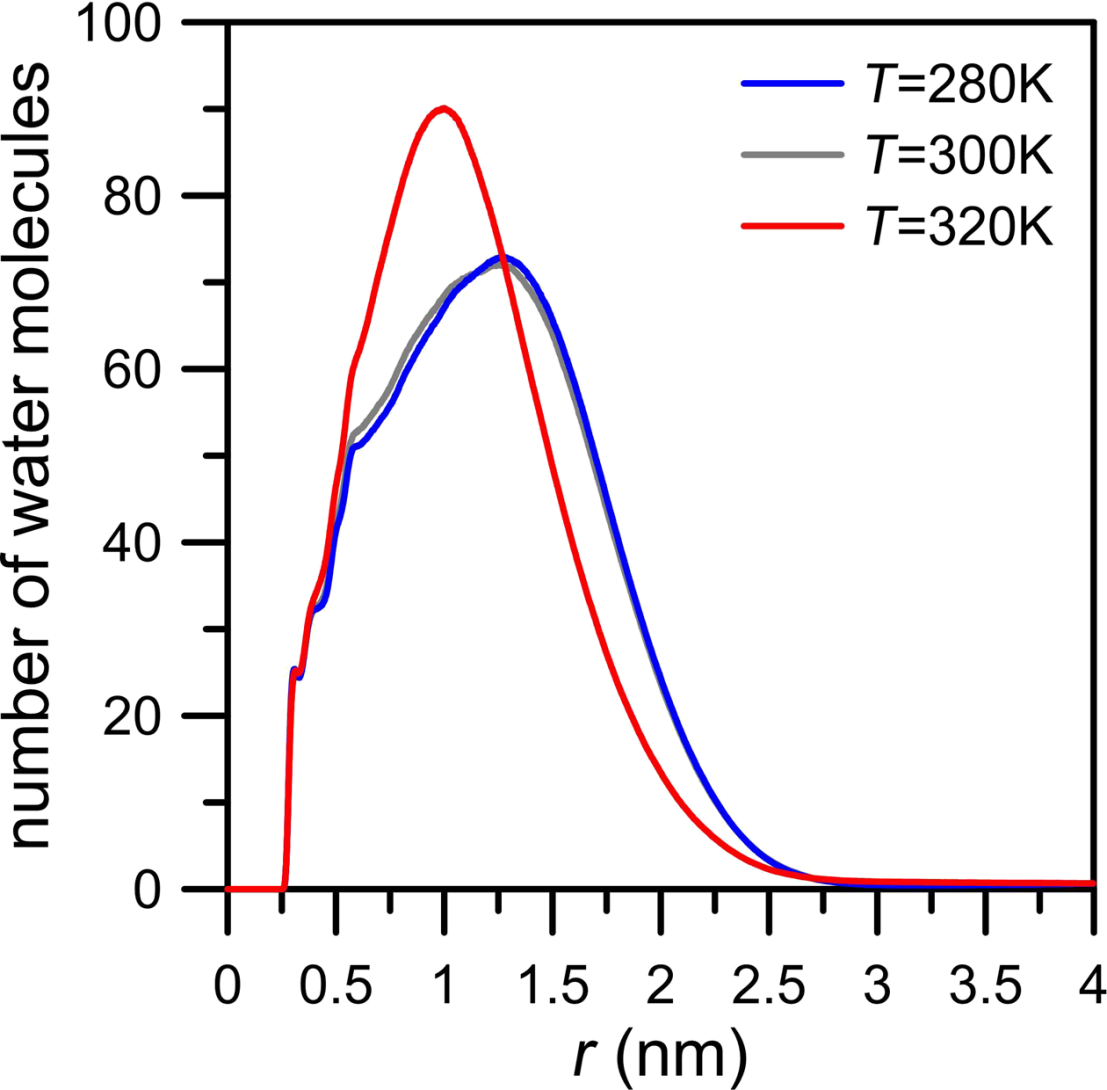
The temperature effect on the number of water molecules as a function of the distance of O_w_ atoms from the center of the rim of an NN nanopore (defined as the geometric center of nitrogen atoms in the nanopore rim) calculated for a CH_4_ + N_2_ gaseous mixture containing 200 water molecules. Other notes are the same as in Figure 4.

The above considerations show that the inhibiting effect of water on gas permeation is strong when the nanopore edge includes strongly electronegative atoms such as nitrogen. The next factor which should be taken into account in order to better understand these phenomena is the influence of the temperature on simulation systems, in particular the question of how the temperature influences the behavior of the water molecules. For this purpose we decided to compare the rate of permeation through an NN nanopore at three different temperatures: 280, 300 and 320 K. The results of these simulations are showed in Figure 11. In general, the number of passings of mixture components at 280 and 300 K are similar. A completely different behavior can be observed in the case of water permeation at 320 K. Figure 11, top panel, shows a sharp increase of the number of water molecules in the permeate area at a time of about 2 ns. However, it may falsely suggest a change of the role of water in gas separation. A similar, though less clear, effect can be observed for this simulation system at 300 K. Figure 7, Figure 8 and Figure 10 dispelled doubts and proved the presence of a water drop in the center of nanopore. The observed rapid increase of the number of water molecules in the permeate area (Figure 11, top panel) is exactly the same case. Figure 12 proves the existence of the water drop in the center of the nanopore. The increase of temperature of the system boosts the kinetic mobility of molecules and hence the gas pressure in the retentate area. As a result, at the initial stage of simulations, the increase of temperature causes an increase of pressure difference between the retentate area and the permeate area. However, the temperature of 320 K is still too low to break hydrogen bonds between water molecules and between water and nitrogen atoms located in the nanopore rim. Summing up, the rapid increase of the number of water molecules in the permeate area observed at 320 K (Figure 11) does not mean that the water drop in the NN nanopore is destroyed. It still exists but because of the higher pressure gradient between retentate area and permeate area the drop is partially sucked to the retentate area. Such a conclusion is proved by Figure 12 where the NWM function determined from simulations at 320 K is a bit narrower and its maximum is visibly higher than the NWM at 280 K and 300 K. It means that the temperature increase may make the water drop more dense and move towards the center of nanopore.

**Figure 13:**
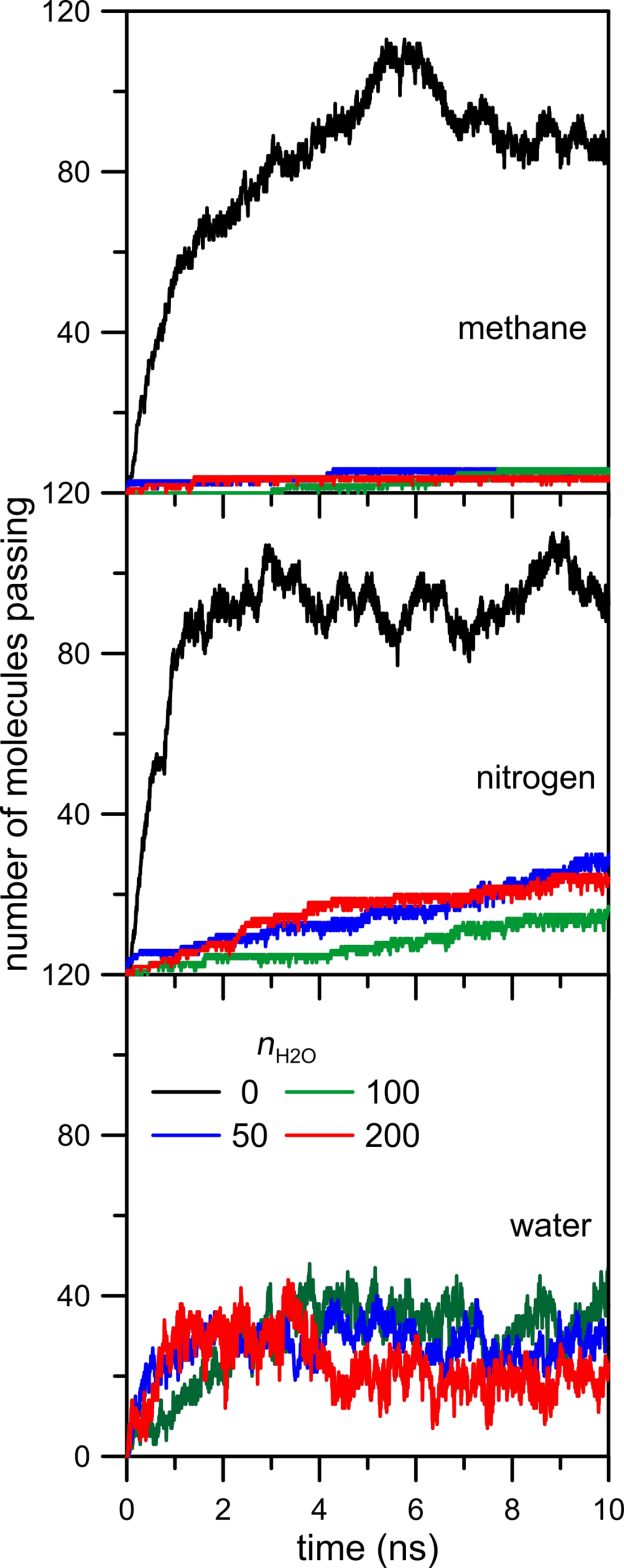
The influence of the number of water molecules present in the gaseous mixture on the number of CH_4_ and N_2_ molecules passing through an NN nanopore at 300 K.

Another effect worth considering is the influence of the number of water molecules present in the gaseous mixture on gas permeation. For this purpose we performed additional simulations of CH_4_ + N_2_ separation with an NN nanopore at 300 K adding 50 and 100 water molecules. Figure 13 shows a negligible impact of the NWM on gas separation. Even when the separated mixture includes only 50 water molecules the rate of methane and nitrogen permeation visibly decreases. The shapes of the curves in Figure 13 determined for mixtures with 50, 100 and 200 water molecules are almost the same. It means that the blocking effect caused by water is very strong.

Making sure the temperature and the number of water molecules in gaseous mixture do not have much impact on the blocking effect caused by water in simulated systems, it is worth investigating the nature of the interactions between water molecules and nanopore rim. The relative stability of hydrogen bonds formed between water and nitrogen atoms that are utilized to passivate nanopores can be studied using the history-independent correlation function *c*(*t*) of the formation of hydrogen bridges [23]:

[1]
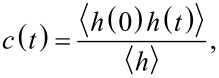


where *h*(*t*) denotes the specific binary function: If the hydrogen bond between a particular donor/acceptor exists then *h*(*t*) = 1 and otherwise *h*(*t*) = 0. The expressions in angle brackets are time-averaged. *c*(*t*) can be interpreted as a conditional probability that the hydrogen bond is intact at time zero. The integral of the correlation function *c*(*t*) leads to the approximation of an average hydrogen-bond lifetime:

[2]
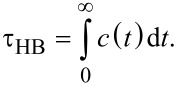


**Figure 14:**
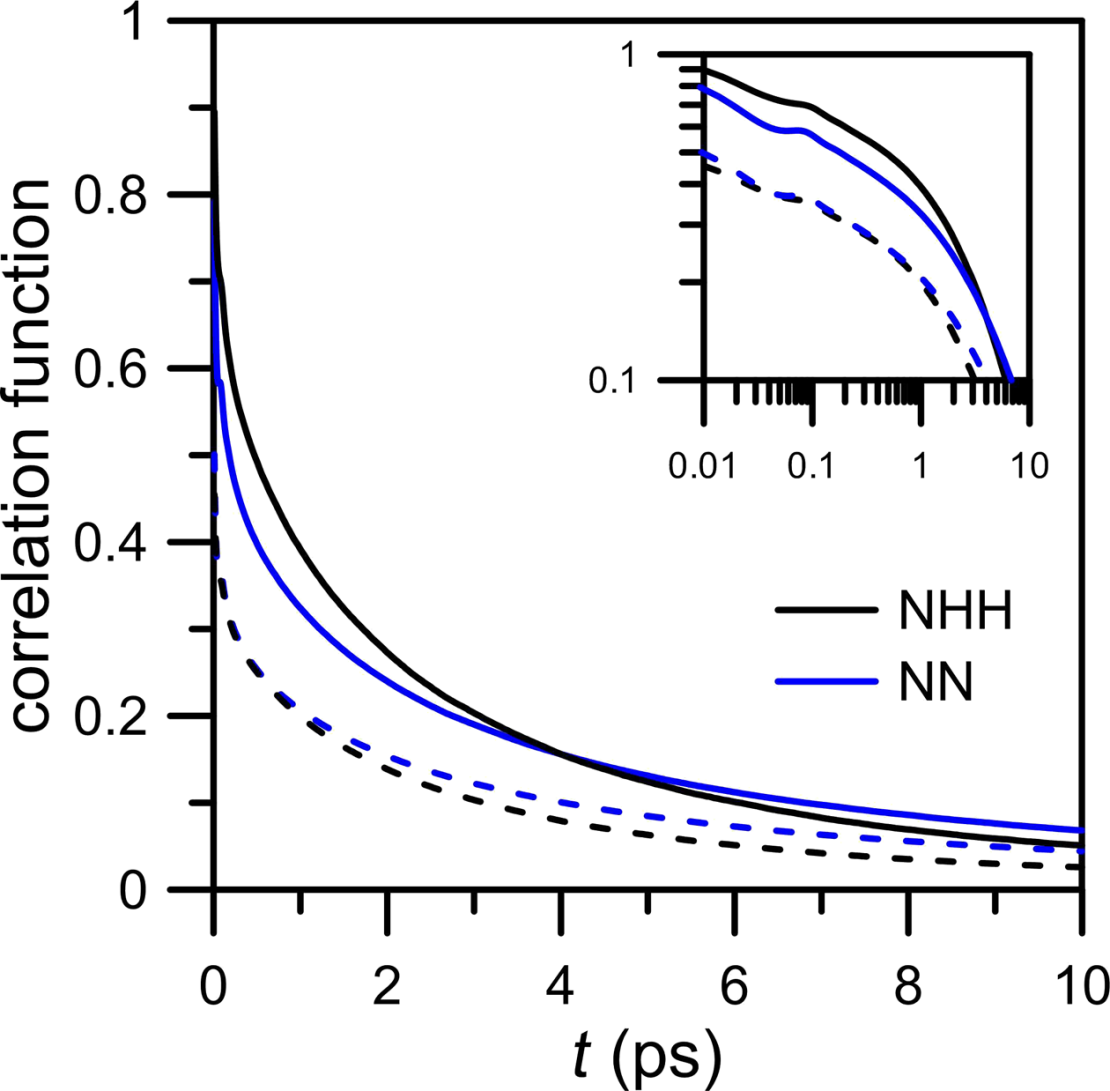
Hydrogen-bond correlation functions for hydrogen bridges between water and nitrogen atoms located in the nanopore rims calculated for separation systems containing 200 water molecules. The inset panel shows the log–log plot of the same data. The solid lines are autocorrelation functions corresponding to water–nitrogen pairs between which at least one hydrogen bond was made during simulations, whereas the dashed lines are AC functions calculated using all possible water–nitrogen pairs, i.e., even those between which there was no hydrogen bond.

Through calculating τ_HB_ for hydrogen bonds between water and nitrogen atoms present in NHH and NN membranes, we can compare their relative stability. To calculate the correlation function *c*(*t*), the binary functions *h*(*t*) of all possible donor/acceptor pairs (hydroxy groups originating from water and nitrogen atoms located at the nanopore rim) were determined. Next, for individual donor–acceptor pairs the correlation functions were first calculated and then averaged. Thus, the history-independent correlation functions shown in Figure 14 are averaged curves of many trajectories: In the case of an NHH nanopore and a gaseous mixture with 200 water molecules there were 1200 trajectories (three nitrogen atoms and 400 hydroxy groups), while for an NN nanopore there were 3600 trajectories. From a technical point of view two cases can be considered during calculations: the first case in which *h*(*t*) were considered for all donor–acceptor pairs and the second case in which the number of trajectories was reduced by those in which no binding was observed.

In the second case we can see that the hydrogen bonds between water and nitrogen atoms located in the NHH membrane are more stable than in NN membrane system. As an example, Figure 14 shows that the probabilities that a particular hydrogen bond is undisturbed in a period of 2 ps are 0.3 and 0.25 for NHH and NN membranes, respectively. This result is surprising. Nitrogen atoms in an NHH nanopore have a less negative charge than the average charge of nitrogens in an NN nanopore. It is possible that hydrogen bonds between water and nitrogen from NHH membrane can be stabilized due to the electrostatic interactions between water oxygen O_w_ and hydrogen atoms with positive charge presented in the nanopore. Taking into account all possible trajectories (case 1), the conclusions delivered by correlation functions *c*(*t*) are in line with the expectations. Hydrogen bonds in the NN nanopore are more stable than in the NHH membrane system. These different results are the evidence for weak water diffusion in the investigated systems.

The history independent correlation function *c(t)* can be used to determine the reactive flux correlation function [24]:

[3]
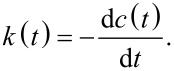


The function above is the average of the integrated flux for those trajectories where the bond is broken at a later time *t* [24]. Because the probability that in a large system at equilibrium a particular donor–acceptor pair is hydrogen-bonded is small, the correlation function *c*(*t*) relaxes to zero and therefore *k*(*t*) describes the rate of relaxation to equilibrium. It is worth noting that at long times the reactive flux correlation function is invariant with respect to the specific definition of a hydrogen bond. At initial times its behavior provides the information about the mechanism of hydrogen bond formation but it is sensitive to the definition of hydrogen bond [24].

**Figure 15:**
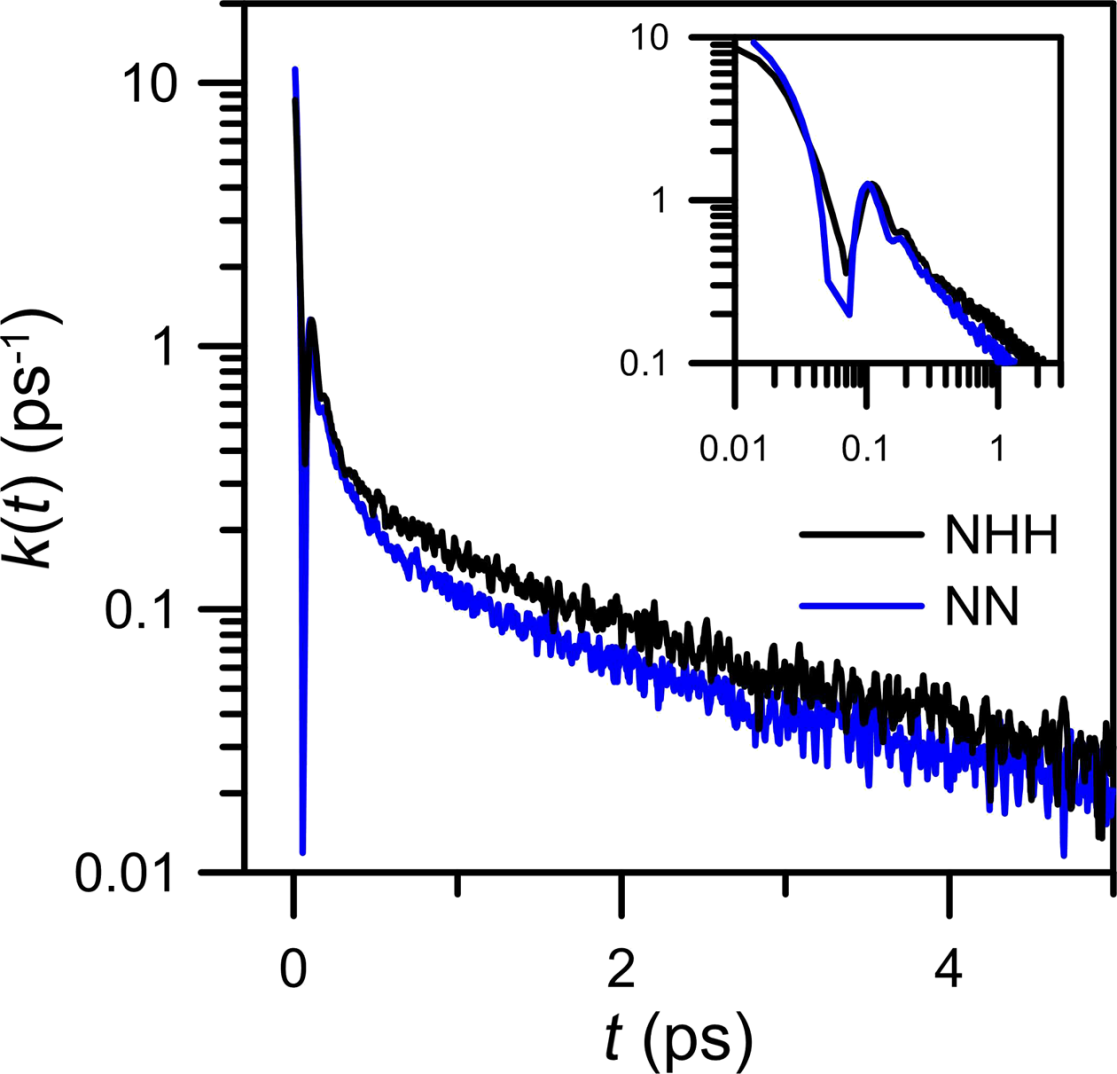
Semi-logarithmic plot of the reactive flux correlation functions *k*(*t*) for hydrogen bridges between water and nitrogen atoms calculated for NHH and NN separation systems. The inset panel shows the log–log plot of the same data.

The reactive flux correlation functions *k*(*t*) determined numerically using *c*(*t*) (case 1) are presented in Figure 15. At short times, *k*(*t*) for NHH and NN membranes are similar. The log–log plots in the inset panel of Figure 15 shows at short times rapid changes of *k*(*t*) from its initial values. This indicates many recrossings in and out of the bonding region at a time scale of less than 0.08 ps. Following Luzar and Chandler [24], such a rapid decrease of the reactive flux correlation function at the initial times can be interpreted as librations. Next, in the range from 0.08 to 0.1 ps the increase of *k*(*t*) is caused by the inter-oxygen vibrations. Beyond this transient period of time both curves decay monotonically. A faster decay of *k*(*t*) calculated for the NN membrane indicates that the time necessary to reach the equilibrium state is shorter.

**Figure 16:**
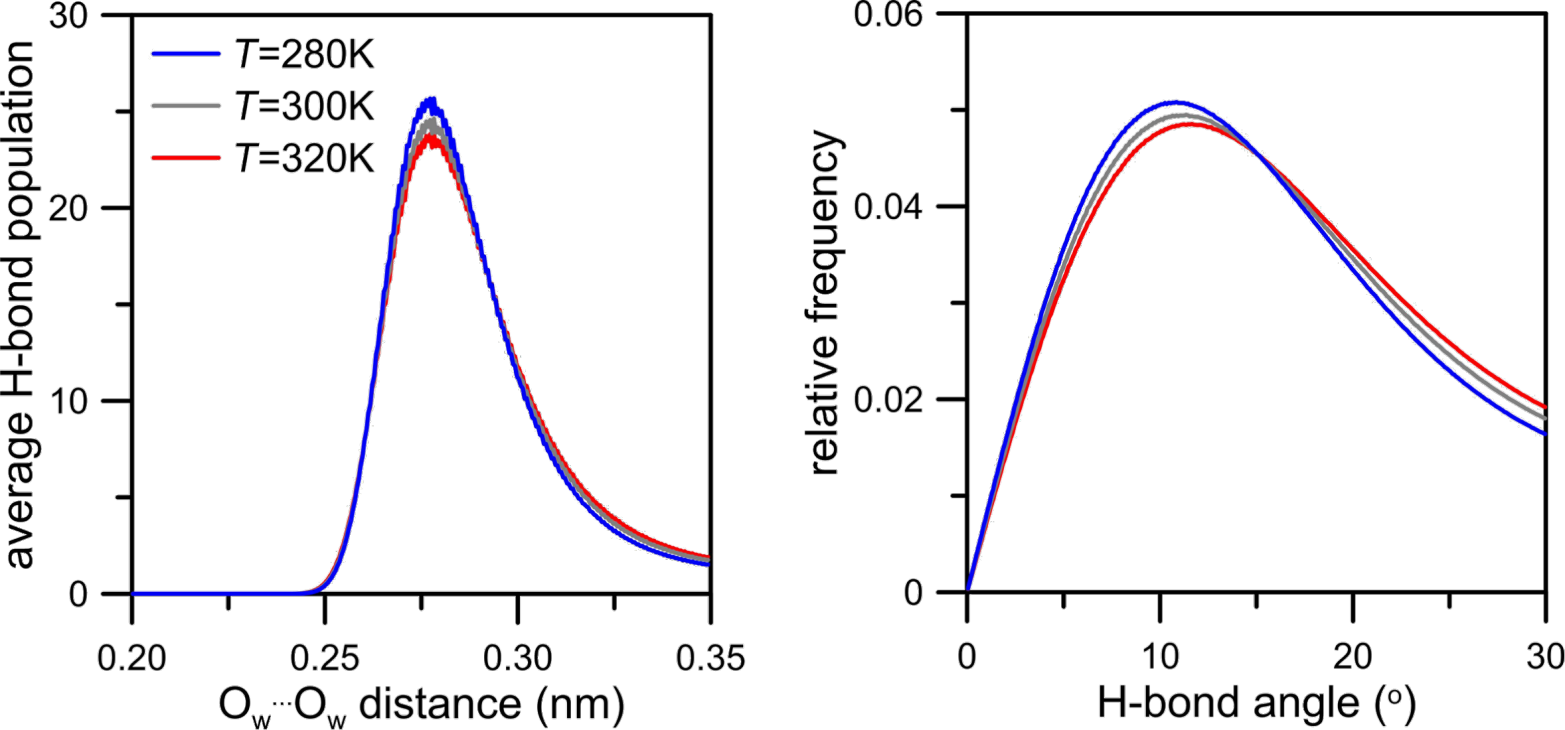
Temperature influence on (a) the hydrogen bond distance and (b) the angle distribution in water for an NN membrane system and a CH_4_ + N_2_ gaseous mixture containing 200 water molecules.

The hydrogen bonds between water and nitrogen atoms from the nanopore rims are not the only bonds in the investigated systems. The observed water drop in the close vicinity of the center of a nanopore is formed through the hydrogen bonds between water molecules. Because the electronegativity of oxygen is even higher than that nitrogen, hydrogen-bond interactions in water seem to be stronger than between nitrogen and –O_w_H. Figure 16 shows the distribution of hydrogen bonds geometries in water calculated for a simulation system including an NN membrane and a CH_4_ + N_2_ gaseous mixture containing 200 water molecules. The geometric criteria for water–water hydrogen bonds are the same as for the ones between water and nitrogen: the cut-off distance *R* = 0.35 nm and the bond angle 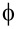 = 30°. It can be seen that most hydrogen bonds in water are, following the Jeffrey classification [25], moderate and mostly electrostatic. A significant part of hydrogen bonds in water fulfills the cut-off criteria: The most probable bond distance and angle are 0.27 nm and 12°, respectively. The increase of simulation temperature causes an increase of kinetic mobility of the molecules and hence the distance and angle distributions are slightly wider.

**Figure 17:**
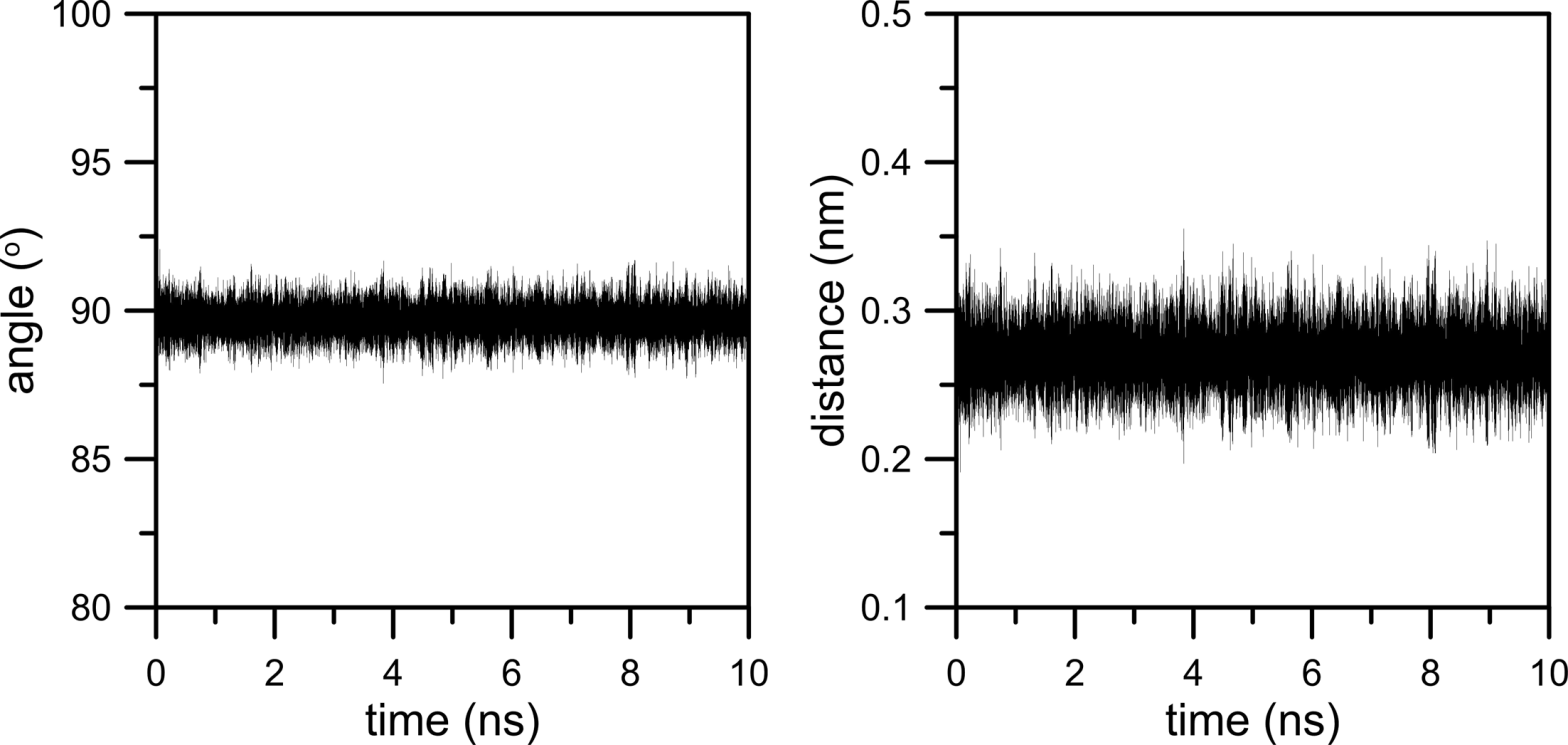
Evolution in time of the average angle between four vectors designated by four carbon atoms in every graphene corner and the nearest nitrogen atoms in the nanopore rim and the normal to the plane that includes four fixed carbon atoms in every graphene corner (first kind of deviation, left plot). The right plot presents the time dependence of the distance between the center of nanopore and that plane. Data were determined from the simulation trajectory of CH_4_ + N_2_ + 200 H_2_O at 300 K.

**Figure 18:**
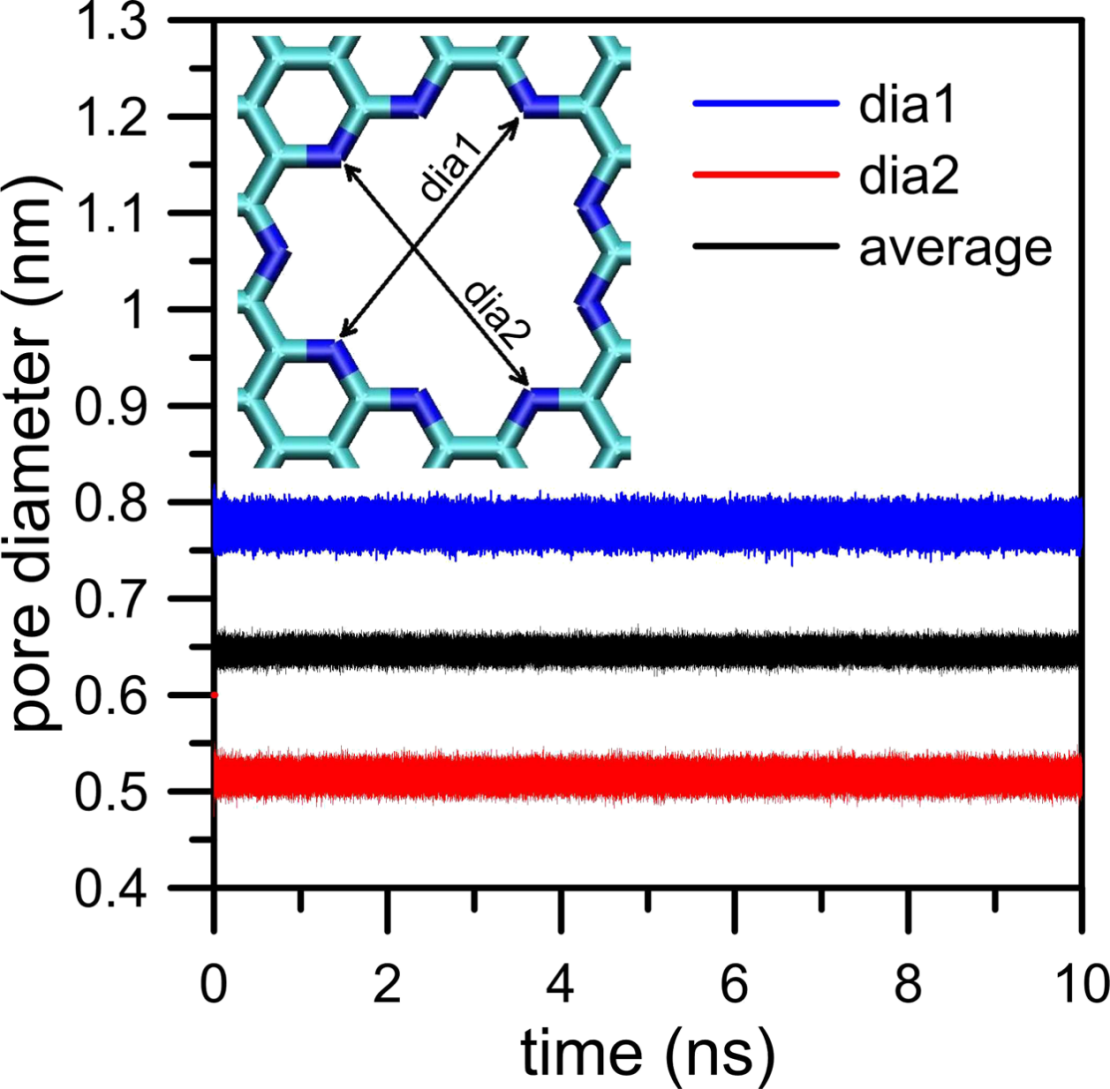
Time evolution of the second kind of deviation. Data were determined from the simulation trajectory of CH_4_ + N_2_ + 200 H_2_O at 300 K.

While describing the simulation model we assumed that the graphene surface is not completely flat and there may be some wrinkles and fluctuations in the graphene membrane. Because this effect can influence gas permeation as well as, we made an effort to investigate it more precisely. For this reason we considered two aspects of membrane deformation: the deviation of the graphene sheet from the plane (the first kind of deviation) and a change of the average pore diameter in time (the second kind of deviation). To investigate the deviation of the graphene surface from the plane for the case of an NN membrane we defined four vectors designated by four carbon atoms in every graphene corner and the nearest four nitrogen atoms in the nanopore rim. Then, the first kind of deviation can be measured by the time evolution of the average angle between these four vectors and the normal to the plane that includes four fixed carbon atoms in every corner of the graphene sheet. Moreover, the time evolution of the distance of the center of the nanopore (defined as the geometric center of nitrogen atoms located in the nanopore rim) from the plane is worth observing. The second type of the deviation can be measured as the average distance between two pairs of nitrogen atoms that are located on the opposite sides in the nanopore (e.g., the same nitrogen atoms used to define vectors in the case of the first kind of deviation). Figure 17 and Figure 18 illustrate the level of membrane deformation.

It can be seen that during simulations the membrane undergoes vertical and horizontal deformations. The first kind of deviation illustrated in Figure 17 shows the shift level of the nanopore in the *z*-dimension. As a result, the membrane may have a shape of a hill or a dale. The average angle that reflects the elevation angle takes values of about ±2°. Moreover, in relation to the completely flat membrane the nanopore center can move along the *z*-dimension by approximately 0.28 nm.

The second kind of deviation is illustrated in Figure 18. It can be observed that the nanopore undergoes horizontal strains (deformations in *x*- and *y*-dimension). The nanopore is not a perfect circle and becomes stretched. As a result, the average diameter varies in the range of 0.64 nm ± 0.02 nm.

A separate question is to which extent membrane deviations affect the hydrogen bonds between water and nitrogen atoms in the pore rim. The variance in the elevation angle is about 2.2%. Because the *z*-dimensions of retentate and permeate areas are 8 nm also the vertical shift of the nanopore seems to be negligible (ca. 2.9%). Similarly, while taking into account the second kind of deviation the variance in pore diameter is about 3.1%. Thus, fortunately, in relation to the size of the simulation box it seems that fluctuations in the shape of graphene membrane are rather small and can be neglected. Nevertheless, this problem will be investigated in future studies.

## Conclusion

Using molecular dynamics it has been demonstrated that the natural gas separation with functionalized graphene membranes can be very sensitive to the presence of water. The increase of the number of highly electronegative atoms such as nitrogen in the nanopore rim creates the opportunity to form hydrogen bridges with mixture components and then influences their selectivity of separation. Moreover, the presence of water in the separation system can decrease the membrane permeability. Due to the presence of hydrogen-bond acceptors in the nanopore, water molecules gather in the surroundings of the permeation path. As a result, the nanopore may be blocked by a water drop and the rate of permeation can decrease abruptly. These conclusions are confirmed by radial distribution functions as well as history-independent autocorrelation functions. The stabilities of hydrogen bridges in the nanopore rim are similar for both functionalized nanopores. The increase of the number of nitrogen atoms in the nanopore causes the decrease of the HB lifetime. The reactive flux autocorrelation function calculated for the NN membrane relaxes to zero faster than that calculated for the NHH membrane.

The studies presented here provide purely theoretical insights into the gas separation with nanoporous graphene membranes. Thus, to make results applicable in practice experimental works are needed. The authors hope that this work may yield new concepts to perform such studies.
